# Editorial: Innovative approaches and therapeutic perspectives for early-onset neurodevelopmental disorders: from bench to bedside

**DOI:** 10.3389/fnins.2024.1370030

**Published:** 2024-02-27

**Authors:** Barbara Bardoni, Maria Vincenza Catania, Viviana Trezza

**Affiliations:** ^1^Inserm U1323, CNRS UMR7275, Institut de Pharmacologie Moléculaire et Cellulaire (IPMC), Valbonne, France; ^2^Institute for Biomedical Research and Innovation, National Research Council (CNR-IRIB), Catania, Italy; ^3^Department of Science, Roma Tre University, Rome, Italy

**Keywords:** neurodevelopmental disorders, Fragile X Syndrome, Down Syndrome, Rett Syndrome, Shank3a, Prader Willi Syndrome, Schaaf-Yang Syndrome, Down Syndrome Regression Disorder

The topic “*Innovative approaches and therapeutic perspectives for early-onset neurodevelopmental disorders: from bench to bedside*” stands as a crucial exploration into the realm of neurodevelopmental disorders (NDDs). Organized into six insightful reviews and seven groundbreaking original research papers, this Research Topic is a witness of the dynamic and evolving nature of research in the field.

Palmieri et al. lead the charge by not only providing a comprehensive overview of the state-of-the-art treatment for Rett Syndrome (RTT) but also delving into the potential application of cutting-edge therapies utilizing molecular delivery through nanoparticles. This forward-thinking approach opens up new possibilities for the treatment of RTT, a severe form of NDD.

Similarly, Tempio et al. embarked on a journey to unravel the complexities of Fragile X Syndrome (FXS) by framing it as an interneuronopathy. Their proposal to reintroduce functional interneurons into the brains of FXS patients, based on recent findings regarding the isolation of FXS interneurons and alterations in the Meis2-expressing interneuronal class (Castagnola et al., 2020), adds a novel dimension to therapeutic exploration.

Bertocchi et al. focused their review on the challenges associated with generating and validating preclinical models of developmental and epileptic encephalopathies (DEEs). Their objective is to identify new molecular targets specific to these syndromes and to gain a better understanding of associated comorbidities, such as behavioral and cognitive deficits.

Desprez et al. contributed to the collective knowledge by updating information on dihydropyrimidinase-like (DPYSL) proteins. They shed light on the role of these proteins in synaptic processing during later stages of neurodevelopment and their potential contribution to the pathophysiology of autism spectrum disorders (ASD) and intellectual disability (ID).

Dobrigna et al. provided a comprehensive overview that navigates through the intricate molecular changes in group I p21-activated kinases (PAK1, 2, and 3) and their implications across a broad clinical spectrum of NDDs. The authors underscore the importance of understanding different PAK mutations for the development of personalized treatments.

The review by Li et al. takes a unique perspective by analyzing the effects of various physical activities in children with Attention Deficit Hyperactivity Disorder through a network meta-analysis. Their emphasis on tailoring physical activity based on individual symptom severity brings attention to the personalized nature of interventions.

Turning to original research articles, three delve into Down syndrome, the most common form of genetic intellectual Disability (ID).

Bonne et al. investigated an uncommon neurodevelopmental regression termed Down Syndrome Regression Disorder, distinct from ASD, with an unknown etiology. Symptomatic therapeutic interventions proved ineffective and poorly tolerated in the four analyzed patients (Bonne et al.). In contrast, etiological treatments, such as anti-inflammatory drugs and corticosteroids, resulted in partial or substantial recovery in all cases.

Thomazeau et al. endeavored to unravel the intricate synaptic underpinnings of prefrontal cortex (PFC) dysfunction in Down Syndrome (DS). Building on a prior study involving mBACtgDyrk1a mice, where synaptic plasticity deficits within the PFC were observed (Thomazeau et al., 2014), their focus shifted to another DS model – the Ts65Dn mice. These mice exhibit the overexpression of several genes, including Dyrk1a, a key gene in the pathophysiology of DS. In this study, Thomazeau et al. identified alterations in the intrinsic properties of PFC layer V/VI pyramidal neurons in Ts65Dn male mice. Notably, they discovered the absence of long-term depression, while synaptic or pharmacological long-term potentiation remained fully expressed (Thomazeau et al.).

Conan et al. employed a multifaceted approach, combining genetic and drug screenings utilizing a cellular model that overexpressed CYS4, the homolog of Cystathionine beta synthase (CBS) in Saccharomyces cerevisiae. Their goal was to gain further insights into the molecular mechanisms governing the regulation of CBS, a pivotal protein underlying DS pathology along with Dyrk1a (Panagaki et al., 2022). The study shed light on the significance of Akt/GSK3β and NF-κB pathways in regulating CBS activity and expression.

Shovlin et al. employed a unique approach to pinpoint molecular biomarkers and surrogate endpoints for RTT. They utilized RNA sequencing to assess differential gene expression in whole blood samples from participants in the phase I mecasermin trial. Mecasermin, a recombinant human IGF-1, had previously shown success in pre-clinical tests with RTT mouse models. The analyses identified gene expression profiles linked to the severe breathing phenotype and its improvement following mecasermin administration in RTT. This study led the authors to a significant conclusion, indicating the involvement of inflammatory/immune pathways and IGF-1 signaling in treatment response. Consequently, it steers future investigations toward a novel direction in understanding the pathophysiology of RTT.

In Magel2-knockout (KO) mice, a model of Schaaf-Yang Syndrome, the dysregulation of oxytocin receptors (OXTR) in the hippocampus of adult male mice is normalized through oxytocin (OXT) treatment at birth, resulting in the rescue of autistic-like behavior and cognition in adulthood (Bertoni et al., 2021). Gigliucci et al. analyzed both male and female Magel2-KO mouse brains at different life stages, concluding that OXTRs undergo region-specific modifications related to age, sex, and postnatal OXT treatment. These findings provide valuable insights for tailoring precisely-timed OXT-based therapeutic strategies in Schaaf-Yang Syndrome patients (Gigliucci et al.).

Bouquier et al. introduced a groundbreaking transgenic mouse line, the Shank3Venus/Venus knock-in mouse, enabling the monitoring of the endogenous expression of the major Shank3 isoform in the brain. Mutations in this isoform cause a form of ASD. The study revealed a developmental delay in the brain expression of the Venus-Shank3a isoform in Shank3Venus/ΔC mice compared to Shank3Venus/+ control mice (Bouquier et al.). This innovative approach serves as a powerful tool to study endogenous Shank3a expression under physiological conditions and in ASD, facilitating isoform-specific investigations of endogenous Shank3 proteins.

Prader-Willi disorder (PWS), a NDD characterized by growth delay, hypogonadism, narcolepsy, lack of satiety, compulsive eating, and mild to moderate cognitive impairment, was the focus of the study by Louveau et al.. They examined the response to topiramate in 24 patients affected by different genetic causes of PWS, including deletion or uniparental disomy (UPD) in a region of chromosome 15. The study revealed that topiramate was less effective and less tolerated in UPD cases compared to deletion cases. Interestingly, despite these differences, patients with deletions exhibited less severe clinical features compared to those with UPD (Louveau et al.). The study suggests the relevance of a pharmacogenomic-based approach for studying PWS.

In conclusion, this thematic Research Topic offers a sweeping overview of a rapidly evolving research field, encapsulating various NDDs. The articles collectively tackle crucial issues, including phenotypic complexity, the role of sex as a biological variant, challenges in developing validated models, complexities in drug development, and the imperative need for innovative delivery methods and cell-based treatments and the pharmacogenomics as new approach to unravel new pathophysiological elements. These insights not only contribute significantly to our current understanding but also lay the foundation for future, more targeted explorations within the expansive realm of NDDs.

## Author contributions

BB: Writing—original draft. MC: Writing—review & editing. VT: Writing—review & editing.

## References

[B1] Bertoni A. Schaller F. Tyzio R. Gaillard S. Santini F. Xolin M.. (2021). Oxytocin administration in neonates shapes hippocampal circuitry and restores social behavior in a mouse model of autism. Mol. Psychiatr. 26, 7582–7595. 10.1038/s41380-021-01227-6 34290367 PMC8872977

[B2] Castagnola S. Cazareth J. Lebrigand K. Jarjat M. Magnone V. Delhaye S.. (2020). Agonist-induced functional analysis and cell sorting associated with single-cell transcriptomics characterizes cell subtypes in normal and pathological brain. Genome Res. 30, 1633–1642. 10.1101/gr.262717.120 32973039 PMC7605246

[B3] Panagaki T. Pecze L. Randi E. B. Nieminen A. I. Szabo C. (2022). Role of the cystathionine Œ ≤ -synthase/H2S pathway in the development of cellular metabolic dysfunction and pseudohypoxia in down syndrome. Redox Biol. 55, 102416. 10.1016/j.redox.2022.102416 35921774 PMC9356176

[B4] Thomazeau A. Lassalle O. Iafrati J. Souchet B. Guedj F. Janel N.. (2014). Prefrontal deficits in a murine model overexpressing the down syndrome candidate gene dyrk1a. J Neurosci. 34, 1138–1147. 10.1523/JNEUROSCI.2852-13.2014 24453307 PMC3953590

